# SMAnalyst: A Web Server for Spatial Metabolomic Data Analysis and Annotation

**DOI:** 10.3390/biom15111562

**Published:** 2025-11-06

**Authors:** Zhanlong Mei, Xiaolian Ning, Haoke Deng, Lingyun Chen, Yun Zhao, Jin Zi

**Affiliations:** 1 BGI Genomics, Shenzhen 518083, China; 2 BGI Research, Shenzhen 518083, China

**Keywords:** spatial metabolomics, metabolite annotation, web-based platform, quality control, spatial pattern discovery, differential analysis

## Abstract

Spatial metabolomics is a rapidly advancing field offering powerful insights into metabolic heterogeneity in biological tissues. However, its widespread adoption is hindered by fragmented tools and the lack of comprehensive, open-source GUI software covering the full analytical workflow (quality control, preprocessing, identification, pattern, and differential analysis). To address this, we developed SMAnalyst, an open-source, integrated web-based platform. SMAnalyst consolidates core functionalities, including multi-dimensional data quality assessment (background consistency, intensity, missing values), a comprehensive metabolite annotation scoring system (mass accuracy, isotopic similarity, adduct evidence), and dual-dimension spatial pattern discovery (metabolite co-expression and pixel clustering). It also offers flexible differential analysis (cluster- or user-defined regions). With its intuitive GUI and modular workflow, SMAnalyst significantly lowers the analysis barrier, by providing a unified solution that eliminates the need for tool switching and advanced computational skills. Tested with a mouse brain dataset, SMAnalyst efficiently handles large-scale data (e.g., >14,000 pixels, >3000 ion peaks), effectively filling a critical gap in integrated analytical solutions for spatial metabolomics.

## 1. Introduction

Spatial metabolomics is a rapidly advancing interdisciplinary field that integrates metabolite information with its spatial distribution within tissue samples, offering a powerful approach to elucidate the heterogeneity of metabolic processes in complex biological systems [1,2,3]. Mass Spectrometry Imaging (MSI) techniques, such as Matrix-Assisted Laser Desorption/Ionization (MALDI) [1,4] Desorption Electrospray Ionization (DESI) [5] and Secondary Ion Mass Spectrometry (SIMS) [6], are core technologies driving this field’s development. However, the inherent complexity and vastness of spatial metabolomics data [7], pose significant challenges in areas like data preprocessing, quality control, metabolite annotation, and statistical analysis [8,9]. Therefore, developing user-friendly, functionally comprehensive data analysis platforms is crucial for fully leveraging the scientific potential of this technology and advancing the field.

Despite some progress in certain aspects of spatial metabolomics data analysis, particularly in peak detection and extraction, existing tools still show notable deficiencies in crucial downstream analytical processes, including systematic data preprocessing, comprehensive quality control, and in-depth statistical analysis [10,11]. Table 1 provides an overview of the main functions supported by current software tools for spatial metabolomics analysis and highlights the fragmented nature of the existing ecosystem. While mainstream open-source tools like Cardinal (3.6.3) [12], SmartGate (https://github.com/zhanglabtools/SmartGate) [13] can perform basic data preprocessing, visualization, and clustering, they generally lack robust data quality control modules and powerful metabolite annotation capabilities. Conversely, some more feature-rich tools, such as MSImage (1.2) [14], MSiReader (v1.0) [15], are commercial software. Furthermore, although specialized tools exist for specific stages like data preprocessing [16,17,18], visualization [19,20], pattern analysis [12,21], or identification [22,23,24], their fragmented functionalities significantly raise the barrier to entry, especially for researchers without a strong computational background. This fragmented landscape, as summarized in Table 1, underscores the need for an open-source platform that integrates core functionalities such as data quality control, preprocessing, statistical analysis, and metabolite annotation, within a single, user-friendly environment.

To address these challenges, we developed SMAnalyst (Spatial Metabolomics Data Analyst), an innovative open-source spatial metabolomics analysis software implemented in R (4.4.1). The software integrates four key advanced modules. First, a systematic data QC module assesses dataset quality across multiple dimensions, including background signal consistency, ion intensity distribution, and missing value patterns. Second, a robust annotation and scoring system ensures reliable metabolite identification. This system combines multiple lines of evidence, such as mass accuracy, adduct ion forms, and isotopic distribution matching. Third, SMAnalyst includes multi-dimensional pattern discovery functions. These explore spatial expression profiles at both pixel and metabolite levels. Finally, the platform offers flexible differential analysis options. Users can delineate regions of interest (ROIs) manually or generate them automatically using clustering results. While SMAnalyst focuses on integrating downstream analytical modules, it does not include upstream peak-picking steps, which typically require substantial computational resources and are better handled locally prior to data upload. By integrating these user-friendly functionalities, SMAnalyst significantly enhances the efficiency of spatial metabolomics data analysis. More importantly, it bridges the gap between data and biological interpretation by enabling researchers to comprehensively assess data quality, identify metabolites with confidence, and uncover spatially resolved metabolic patterns that are biologically meaningful. The integration of these modules allows users to trace molecular variations across tissue regions, facilitating hypothesis generation and mechanistic insights into metabolic regulation within the microenvironment.

## 2. Materials and Methods

### 2.1. Data Processing Workflow and Implementation

**Figure 1:**
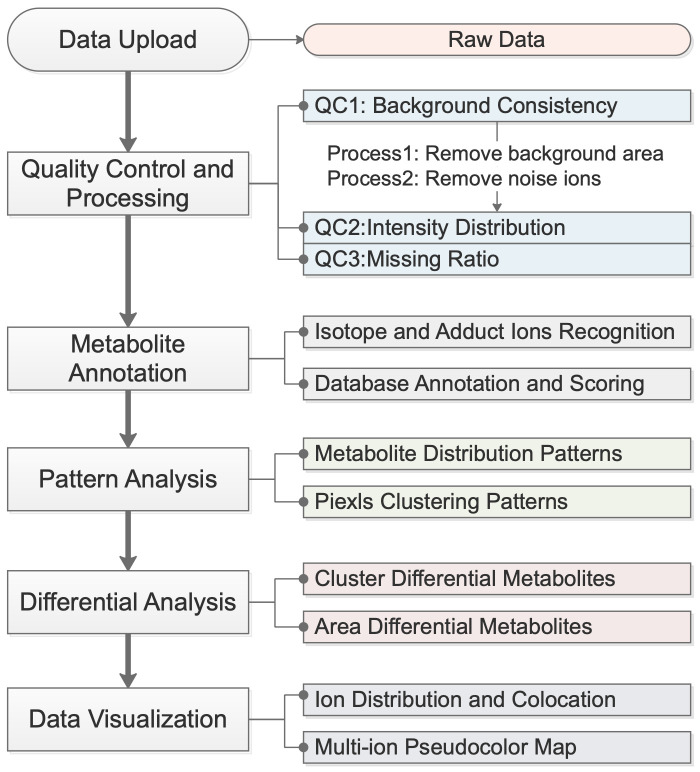
SMAnalyst overall software workflow.

The SMAnalyst analysis workflow is illustrated in Figure 1. It begins with the uploading of compliant spatial metabolomics data. The system then guides users through an integrated analytical process comprising four core modules: (1) Data Quality Assessment and Preprocessing, which evaluates data quality and filters out background pixels and noise ions; (2) Metabolite Annotation, which identifies isotopic and adduct peaks and matches them to metabolite databases with a comprehensive scoring system; (3) Spatial Pattern explore, which explores patterns both at the metabolite (co-expression patterns) and pixel (spatial clustering) levels; and (4) Differential Analysis and Visualization, which supports flexible group comparisons and diverse visualization techniques. Detailed methodologies for each module are described in their respective subsequent sections (Section 2.2, Section 2.3, Section 2.4, Section 2.5, Section 2.6 and Section 2.7).

SMAnalyst is a web-based graphical user interface (GUI) application developed using the R Shiny framework. Users can access its online version via a web browser (https://metax.genomics.cn/app/smanalyst, accessed on 3 November 2025). This version is deployed on a cloud server equipped with a total capacity of 128 CPU cores and 1000 GB of RAM. For user sessions, the system allocates 12 CPU cores and 64 GB of RAM by default, with dynamic resource scaling based on analytical demands. This architecture supports concurrent analysis for over ten users while maintaining performance. The interface is designed with user-friendliness as a core principle, employing an intuitive, step-by-step workflow to guide users through analytical tasks. User-uploaded data are processed only in active memory during the session and are automatically deleted afterward, ensuring users retain full control and rights over their data. For users requiring local deployment, the source code for SMAnalyst v1.0 is open source on GitHub (https://github.com/mzlab-research/SMAnalyst.git, accessed on 16 September 2025), facilitating independent installation and extension.

SMAnalyst requires input data in a Feature Matrix format. The first two columns of this matrix must represent the X and Y spatial coordinates for each pixel, respectively. Subsequent columns correspond to different *m*/*z* values (i.e., detected ion peaks), with the numerical values within the matrix cells representing the intensity of the corresponding ion at that pixel (Supplementary Figure S1). This standardized format ensures SMAnalyst’s compatibility with data generated from various spatial mass spectrometry imaging platforms. Detailed guidelines on how to correctly format input data are available in the software’s tutorial panel (Supplementary Figure S2).

It is important to note that SMAnalyst is a downstream tool and requires data to be converted from raw formats (such as imzML) into this Feature Matrix format using standard upstream processing software (e.g., Cardinal (3.6.3) or vendor software). This initial preprocessing step is necessary due to its high computational demands and is therefore performed prior to uploading data to the SMAnalyst web platform.

### 2.2. Data Processing and Quality Assessment

Upon data upload (Supplementary Figure S3), the pipeline performs data quality assessment and preprocessing. SMAnalyst’s data processing and quality assessment workflow inherits methodologies from our previously developed quality control software, SMQVP 1.0 [27]. Initially, based on the total intensity distribution map of pixels, users can interactively delineate pixel sets representing tissue regions and background regions. The software then visualizes the spectra of the selected background regions to compare spectral consistency across different background areas and calculates the correlation coefficients between spectra to evaluate the spatial consistency of background signals (QC1, Supplementary Figure S4). Next, the average expression levels of each ion in tissue regions versus background regions are compared, ions enriched in tissue (Fold Change > 1) are identified, and a total intensity map of pixels is generated based on these ions. Users can set an intensity threshold to classify pixels as “tissue” or “background”; pixels categorized as background are removed as a preprocessing step (Supplementary Figure S5).

Noise ion identification employs spatial statistical methods. The quadrat test from the spatstat package (3.1-1) [21] is used to assess whether each ion’s spatial distribution conforms to Complete Spatial Randomness (CSR). A “noise score” (defined as the negative logarithm base 10 of the test’s *p*-value) is calculated for each ion. Ions with a noise score below a user-defined threshold are identified as potential noise ions and removed in subsequent analyses (Supplementary Figure S6).

QC2 evaluates signal intensity distribution by generating a spatial distribution map displaying the median ion intensity of each pixel within the sample and a spectral distribution map illustrating the overall pattern of median intensities across all ions (Supplementary Figure S7). QC3 focuses on the issue of missing values, calculating and visualizing two key metrics: (1) the pixel missing ratio (the proportion of undetected ions in each pixel); and (2) the ion missing ratio (the proportion of pixels where each ion was undetected). This helps to identify areas or ions with sparse data coverage (Supplementary Figure S7).

### 2.3. Metabolite Annotation Procedure

**Figure 2:**
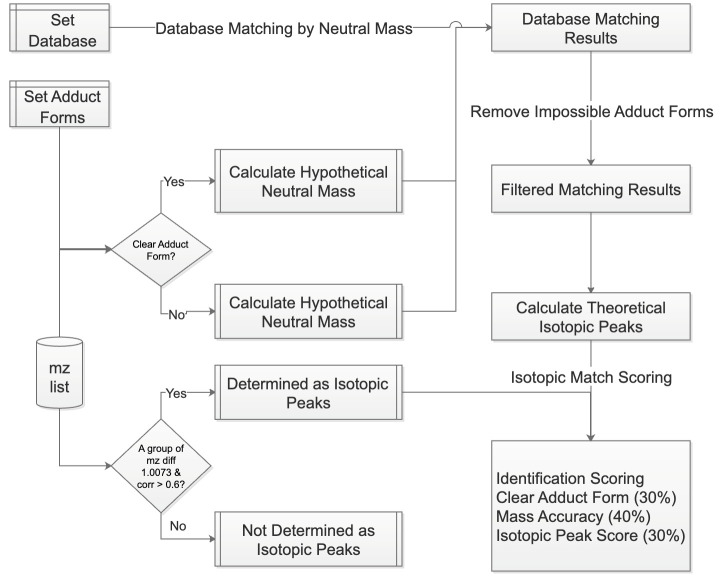
Detailed workflow of metabolite annotation.

SMAnalyst’s metabolite annotation workflow (Figure 2) comprises two core steps: (1) ion peak relationship identification and (2) database matching and scoring. First, the software identifies isotopic peaks and adduct ions within the *m*/*z* list. For isotopic identification, the isotopologues function from the MetaboCoreUtils package (1.12.0) [28] searches for peak pairs conforming to theoretical isotopic mass differences within a user-specified mass error tolerance (ppm). The moran_bv function from the spdep package (1.3-5) [29] then further calculates the spatial correlation of these candidate peak pairs across spatial pixels; if the correlation exceeds a user-defined threshold, they are confirmed as true isotopic peaks (Supplementary Figure S8). Non-monoisotopic peaks are removed and adduct ion identification is performed on the remaining monoisotopic ions. This involves an initial screening for ion pairs matching predefined common adduct mass differences, followed by calculation of their spatial correlation, with final confirmation of adduct ion pairs based on a correlation threshold (the default value is 0.5; a higher threshold may miss true adducts with divergent spatial distributions, while a lower threshold increases the risk of false positive assignments). For identified isotopic and adduct ion pairs, users can select any pair for visualization (Supplementary Figure S8).

In the database matching phase, the first step is to define the metabolite database. SMAnalyst supports two kinds of databases for metabolite annotation: (1) External, high-confidence MS1 mass libraries derived from complementary LC-MS/MS analysis: Users can upload their own databases according to SMAnalyst’s format requirements (specific format details are in Supplementary Figure S9). These are typically LC-MS/MS annotation results from the same sample type as the spatial metabolomics data and are used as an external reference library for matching. We highly recommend prioritizing these self-built, sample-specific databases for more biologically relevant annotations. (2) Internal, built-in public databases: SMAnalyst also incorporates internal, built-in open-source metabolite databases such as HMDB [30], KEGG [31] and LIPIDMAPS [32] for selection. These are used when sample-specific LC-MS/MS data is unavailable.

The matching process differentiates based on whether an ion has a clearly identified adduct form: for ions with a clear adduct form, the calculated neutral mass is directly matched against molecular masses in the database; for ions without a clearly identified adduct form, the neutral mass is calculated sequentially according to a user-specified list of possible adduct forms with mz2mass function in MetaboCoreUtils package (1.12.0) [28] and then matched against the database. The check_ded function from the enviPat package (2.6) [33] is used to check the possibility of the matched molecular formula’s adduct form, eliminating impossible identification results (e.g., a molecule like C7H3F5, lacking oxygen, cannot have an adduct form like [M+H-H_2_O]+). For ions with isotopic peaks, the isopattern function from the enviPat package (2.6) calculates the theoretical isotopic pattern for the matched molecular formula, and then the msentropy_similarity method from the msentropy package (0.1.4) [34] calculates the similarity between the theoretical and actual isotopic patterns. If an ion peak has multiple matching results, the software retains all candidate results for user reference. All matching results are comprehensively scored based on parent ion mass matching accuracy, isotopic peak distribution similarity, and the presence of a clear adduct form. Finally, the number and proportion of ions with identification results, as well as the distribution of *m*/*z* values corresponding to multiple identification results, are summarized (Supplementary Figure S10).

The confidence of metabolite annotation is highly dependent on mass resolving power [35,36,37]. High resolution is critical to resolve isobaric and isotopic interferences that are challenging to distinguish in low-resolution data [35,36]. While SMAnalyst can process data from various instruments, we recommend using high-resolution MSI data to minimize ambiguous identifications.

### 2.4. Pattern Analysis

SMAnalyst’s pattern analysis module offers two complementary strategies to reveal spatial structures within the data. The first strategy operates at the metabolite dimension, utilizing the SpaGene [38] algorithm for spatial expression pattern clustering analysis of metabolic ions (Supplementary Figure S11). This analysis identifies clusters of metabolic ions exhibiting highly similar spatial expression patterns and outputs a list of specific ions contained within each cluster, aiding in the discovery of functionally related metabolite groups. The second strategy operates at the spatial pixel dimension, integrating four clustering methods based on the Seurat package (5.1.0) [39,40]: Seurat-LV (original Louvain algorithm), Seurat-LM (Louvain algorithm with multilevel refinement), Seurat-SLM (Smart Local Moving algorithm), and UMAP-kmeans (Supplementary Figure S12). Users can select any of these algorithms to cluster pixels, aiming to group adjacent pixels with similar overall metabolic profiles into the same category, thereby revealing potential functionally heterogeneous regions within the tissue sample.

### 2.5. Differential Analysis

SMAnalyst supports two flexible strategies for differential metabolite analysis. The first strategy is based on the pixel clustering results from Section 2.4. Users can assign the clustered spatial regions to different biological groups, and the software then compares the expression differences in each metabolic ion between these groups (Supplementary Figure S12). The second strategy is based on user-defined regions of interest (ROIs). Users can interactively delineate multiple spatial regions directly on the tissue imaging map and assign group labels to these regions, after which the software performs inter-group comparisons (Supplementary Figure S13). Both differential analysis strategies utilize univariate statistical testing (Wilcoxon rank-sum test) implemented in Seurat’s [39,40] FindMarkers function. For each comparison, all pixels belonging to the same group are treated as “samples” for that group, and the average expression fold change and significance of difference for each metabolic ion between groups are calculated. To ensure the reliability of the results, *p*-values are adjusted for multiple hypothesis testing using the Benjamini–Hochberg method to control the false discovery rate (FDR), providing a robust identification of differentially expressed metabolites. After analysis, users can online select differential ions of interest and instantly view their spatial distribution maps, facilitating result validation and biological interpretation.

### 2.6. Data Visualization

SMAnalyst also supports various forms of visualization exploration, including generating spatial distribution maps for single ions (single-ion imaging), simultaneously visualizing 2–3 ions (by mapping their intensity values to RGB color channels to create composite pseudocolor images), and performing ion co-localization analysis. For the co-localization analysis, the spatial expression correlation between ions is quantified using Pearson’s correlation coefficient. Specifically, when a user selects a target ion, the software calculates the Pearson correlation coefficient between the spatial intensity of the target ion and that of every other ion in the dataset. It then automatically identifies and displays the spatial distribution images of the six ions exhibiting the strongest positive correlation and the six ions exhibiting stronger negative spatial expression correlations with the target ion, respectively (Supplementary Figure S14). These visualization features provide users with insights into the spatial distribution characteristics of their data.

### 2.7. Test Data

**Figure 3:**
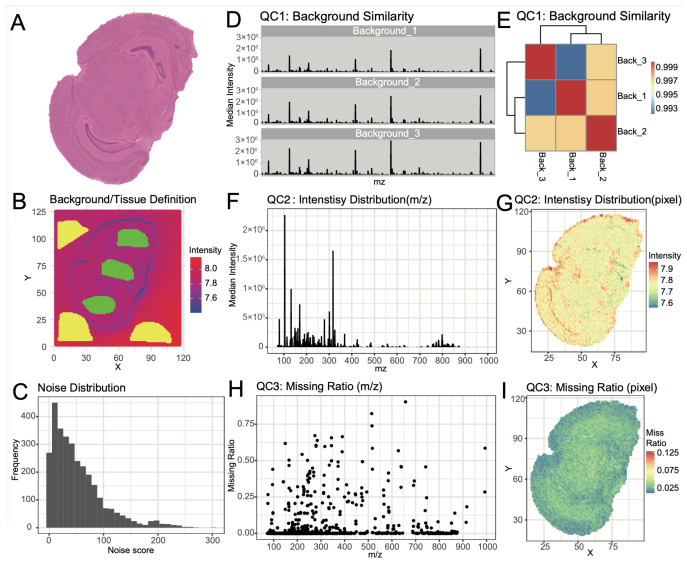
Mouse brain spatial metabolomics data quality control. H&E staining image (**A**); Selection of background and tissue regions (**B**); Noise ion distribution (**C**); QC1: Background region consistency: spectra of background regions (**D**); QC1: correlation of background spectra (**E**); QC2 *m*/*z* intensity distribution (**F**); Pixel intensity distribution (**G**); QC3: *m*/*z* missing rate distribution (**H**); QC3: Pixel missing value distribution (**I**).

To demonstrate SMAnalyst’s functionality and performance, this study utilized two spatial metabolomics data of mouse brain coronal sections collected using the AFAD-ESI platform [5] (positive ion mode). Data acquisition parameters for both two datasets included: spray solvent of acetonitrile and water (80:20 *v*/*v*), AFADESI extraction gas flow rate of 45 L/min, and mass spectrometry detection was performed using a Q Exactive mass spectrometer (Thermo Fisher Scientific Inc., Waltham, MA, USA) with a primary resolution of 70,000. Dataset 1 (anatomical structures shown in Figure 3A H&E staining results) had a pixel size of 100 μm. Dataset 2 had a pixel size of 50 μm. Raw data were processed using Cardinal [12] to generate the feature matrix. The specific workflow included: (1) Peak picking on the total ion image using the ‘diff’ method with an SNR threshold of 6; (2) Peak alignment across pixels with a tolerance of 10 ppm; (3) Filtering to retain peaks present in >10% of pixels; (4) Smoothing the raw data with a Gaussian filter; and (5) Peak area integration based on the filtered peak list. The final feature matrix comprised 14,260 spatial pixels and 3044 unique ion peaks for Dataset 1, and 53,812 spatial pixels and 2654 unique ion peaks for Dataset 2. For reproducibility, the Dataset 1 peak table matrix is available via SMAnalyst’s tutorial panel. To further benchmark the performance of the SMAnalyst cloud platform, we additionally included a public Dataset 3 acquired using an AP-SMALDI source, with a pixel size of 20 μm, comprising 118,604 spatial pixels and 2898 ion peaks.

To support ion peak annotation in spatial metabolomics, we obtained LC-MS/MS annotation results from an adjacent mouse brain slice of Dataset 1 by collecting untargeted metabolomics data using the following method: 25 mg of mouse brain was weighed, precipitant (methanol: acetonitrile: water = 2:2:1) was added. After tissue homogenization, precipitation occurred at −20 °C, and the supernatant was collected by centrifugation and freeze-dried. It was then reconstituted with 50% methanol, and the supernatant was collected after centrifugation for analysis. Chromatographic separation was performed using an ACQUITY UPLC system (Waters) with a BEH C18 column (1.7 μm, 2.1 × 100 mm). Mobile phase: for positive ion mode, water/methanol containing 0.1% formic acid; for negative ion mode, water/95% methanol containing 10 mM ammonium formate. Gradient elution (0–12 min: 2–98% organic phase) was used, with a flow rate of 0.35 mL/min, column temperature of 45 °C, and injection volume of 5 μL. Mass spectrometry detection was performed using a Q Exactive mass spectrometer (Thermo Fisher), with spray voltages of 3.80/3.20 kV for positive modes. Primary MS resolution was 70,000, secondary resolution was 17,500, and stepped collision energy (20/40/60 eV) was applied. Data were processed with Compound Discoverer 3.3 (parent ion mass deviation < 5 ppm), and metabolites were identified through a combined approach using the BGI Metabolome Database, mzCloud, and HMDB, KEGG, and LIPIDMAPS databases.

## 3. Results and Discussion

To systematically evaluate SMAnalyst’s analytical capabilities and integrated workflow, we used the dataset1 for demonstration. This section sequentially showcases the tool’s performance in core aspects, including data preprocessing and quality control, metabolite annotation, spatial pattern discovery, and differential analysis and visualization. Furthermore, we validate the platform’s scalability across larger datasets and demonstrate the impact and robustness of its noise filtering module using both Datasets 1 and 2.

### 3.1. Data Quality Control and Preprocessing

SMAnalyst’s multi-dimensional quality assessment provided a comprehensively evaluation of the mouse brain dataset quality. Assessment of background signal stability showed high spectral consistency across different background regions (Figure 3B,D), with correlation coefficients exceeding 0.99 (Figure 3E), confirming minimal contamination and stable instrument performance. Background pixels were effectively removed using a total ion intensity threshold of 10^7.4^, after identifying tissue-enriched ions (Supplementary Figure S5).

The noise score distribution is shown in Figure 3C. Using the default noise score threshold of 30, which serves as a recommended starting point that users can adjust based on their specific data characteristics, 39% of noise ions were filtered out while retaining 61% of ions for downstream in-depth analysis (Supplementary Figure S6).

Evaluation of data characteristics showed high signal intensity with total cumulative intensity reaching ~7 × 10^7^ (Figure 3F,G), though intensity decreased in the high *m*/*z* region (>800), potentially indicating lower lipid metabolite abundance. Missing value analysis confirmed good data completeness, with most pixels showing <10% missing rate and most ions <5% missing rate across the dataset (Figure 3H,I).

### 3.2. Metabolite Annotation

**Figure 4:**
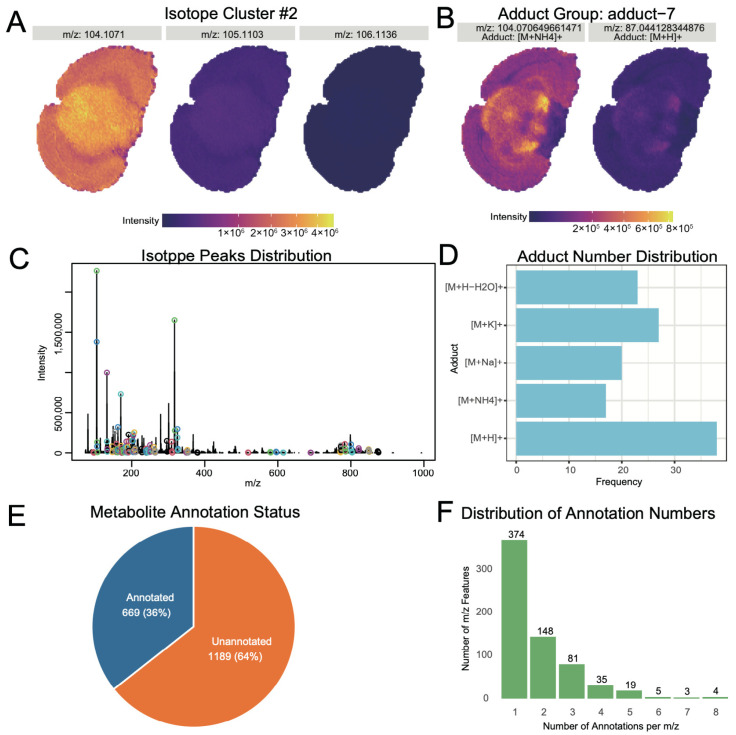
Spatial metabolomics ion annotation. Typical identified isotopic peak pairs (**A**); Overall isotopic peak distribution (**B**); Typical identified adduct ion pairs (**C**); Overall distribution of adduct ion forms (**D**); Proportion of ion peaks with annotation results (**E**); Distribution of one-to-many matching results for ion peaks (**F**).

Metabolite annotation forms the foundation for subsequent biological interpretation. The first step in metabolite annotation is the identification of isotopic peaks and adduct ions in the mass spectrometry data. Identified isotopic peaks accounted for 5.92% of the total ion peaks (Supplementary Figure S8). Figure 4A displays a typical isotopic peak cluster image, where the monoisotopic intensity is higher than non-monoisotopic peaks, and their spatial distributions are similar. Figure 4C further shows that high-intensity ions generally possess isotopic peak clusters. For adduct ion identification, we considered common adduct forms ([M+H]^+^, [M+K]^+^, [M+Na]^+^, [M+NH_4_]^+^, [M+H-H_2_O]^+^), identifying adduct ions comprising 4.19% of the total ions (Supplementary Figure S8). Figure 4B illustrates a typical adduct ion image, while Figure 4D shows the distribution of ion numbers with different adduct forms. Besides [M+H]^+^, the proportions of [M+Na]^+^ and [M+NH_4_]^+^ were also relatively high, a pattern consistent with typical spatial metabolomics data [23].

During the data identification phase, LC-MS/MS annotation results from mouse brain, comprising 1269 metabolites with identification levels 1–3, were selected as a self-built library for metabolite annotation (Supplementary Figure S10). These 1269 metabolites span 29 major molecular classes, with the most abundant categories being Amino acids, peptides, and analogs (14%), Glycerophosphocholines (10%), and Carbohydrates and carbohydrate conjugates (6%). By matching the neutral mass of spatial metabolomics ions with the self-built library and integrating similarity scores for isotopic distribution and adduct information for comprehensive scoring, we successfully identified 669 ions (Figure 4E). Among these, 374 ions had a single annotation result, 148 ions had two annotation results, and 147 ions had three or more annotation results (Figure 4F).

To validate the performance of our annotation workflow, we compared SMAnalyst with MSIannotator [23], using the same spatial metabolomics feature matrix and the same library. MSIannotator identified 655 ions in total, with 362, 146, and 157 ions having one, two, and multiple annotation results, respectively (Supplementary Figure S15A,B). Notably, the overlap between ions with single annotations in both tools reached 91% (Supplementary Figure S15C), demonstrating high concordance and validating the reliability of SMAnalyst’s annotation pipeline.

### 3.3. Spatial Pattern Discovery

**Figure 5:**
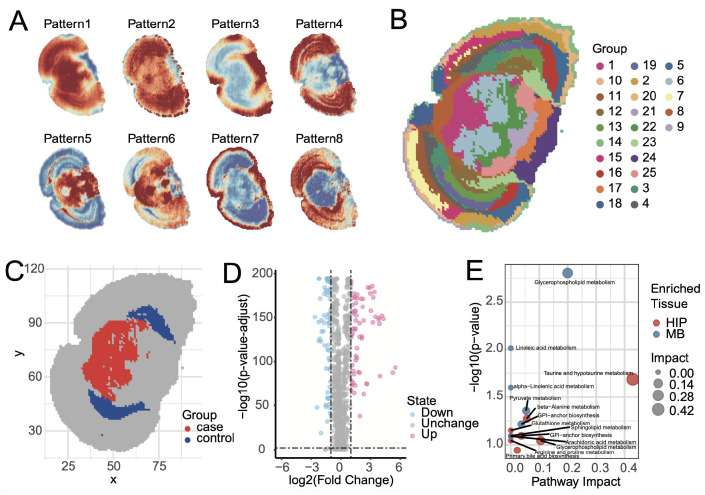
Spatial pattern identification and differential analysis of mouse brain data. Metabolite spatial expression patterns (**A**); Pixel clustering patterns (UMAP–k-means); categorical colors denote cluster identities (see legend). (**B**); Selection of comparison regions based on clustering results; MB (case group, red) comprises clusters 6 and 1, HIP (control group, blue) comprises clusters 23 and 12. (**C**); Differential volcano plot (**D**); Functional enrichment analysis for differential results (**E**).

Elucidating the spatial distribution patterns of metabolites within tissues is a core object of spatial metabolomics. SMAnalyst achieves this through two analytical modules. First, metabolite spatial co-expression pattern analysis revealed eight major metabolite spatial expression patterns within mouse brain tissue (Figure 5A and Figure S11). These patterns clearly demonstrate the synergistic enrichment and regional specificity of metabolites in different brain regions. For instance, Pattern 4 showed high expression primarily in the cerebral cortex and low expression in the midbrain; Pattern 5 was complementary to Pattern 6; Pattern 8 was similar to Pattern 4 but lower in the Entorhinal area; and Pattern 7 displayed unique enrichment characteristics in the tissue edge regions. These synergistic or complementary metabolite expression patterns strongly suggest specific metabolic network activities in different functional brain regions.

Second, the UMAP-kmeans algorithm identified 25 spatially heterogeneous categories at the pixel level (Figure 5B). Comparison of the clustering results with the Allen Mouse Brain Atlas [41] revealed high consistency with known anatomical structures. Major anatomical divisions, such as the cerebral cortex, hippocampus, midbrain, hindbrain, and fiber tracts, were clearly mapped in the clustering results. However, some fine nuclear structures, like the Periaqueductal gray and Superior colliculus, were grouped together in cluster 1 and could not be distinguished. This phenomenon can likely be attributed to the high similarity of their metabolic profiles and relatively gradual spatial transitions between adjacent tissue, which can lead to partial merging of regions at the given spatial resolution and clustering granularity.

### 3.4. Spatial Differential Analysis

Identifying region-specific metabolites is crucial for a deeper understanding of brain region function. SMAnalyst provides flexible analytical tools for this purpose, supporting spatial metabolic differential analysis based on clustering results or manually defined regions of interest (ROI). Figure 5B displays the results of pixel clustering, where each distinct color represents an independent spatial metabolic cluster. To explore metabolic feature differentiation between different functional systems, we compared the midbrain (MB), composed of clusters 6, and 1, and colored red in Figure 5C, with the hippocampal region (HIP), composed of clusters 23 and 12, and colored blue in Figure 5C (Supplementary Figure S12). HIP plays a central role in cognitive functions such as spatial memory and navigation learning, while the selected MB regions primarily involve MBmot for motor output and coordination, and MBsen for sensory signal reception and processing. Differential analysis was performed using the Wilcoxon rank-sum test with Benjamini–Hochberg FDR correction. Metabolites with (1) Fold Change > 2 or <0.5 and (2) FDR-adjusted *p*-value < 0.01 were considered statistically significant. Differential results (Figure 5D) showed that 76 metabolites were significantly upregulated in the MB group, with 27 annotated and 17 uniquely annotated; while 82 metabolites were significantly upregulated in the HIP group, with 29 annotated and 16 uniquely annotated.

Pathway enrichment analysis of uniquely identified differential metabolites using Metaboanalyst [42] revealed that these metabolic features were highly consistent with regional functions. Metabolites upregulated in HIP (e.g., taurine and sphingolipids) were enriched in pathways such as taurine metabolism, sphingolipid metabolism, and glycerophospholipid metabolism (Figure 5E). These pathways are involved in neuroprotection, antioxidant stress, and cell membrane stability, which aligns with the cognitive functional demands of the HIP. Conversely, metabolites upregulated in MBmot and MBsen (e.g., glycerophospholipids and linoleic acid derivatives) were enriched in pathways such as glycerophospholipid metabolism, linoleic acid metabolism, and pyruvate metabolism. These pathways emphasize energy production and cell membrane fluidity, consistent with the high energy consumption in motor regions and rapid signal transmission in sensory regions. Overall, the enrichment patterns of metabolites and pathways validated the biological basis of regional functions.

To further validate SMAnalyst’s analytical performance against established tools, we compared its spatial clustering and differential analysis capabilities with Cardinal [12], using the same mouse brain dataset. When performing pixel clustering to generate 25 spatial categories, Cardinal’s spatial shrunken centroids method produced anatomically recognizable patterns, though with more spatially dispersed clusters compared to SMAnalyst’s UMAP-kmeans results (Supplementary Figure S16A). For differential analysis between midbrain and hippocampal regions defined by Cardinal’s clustering, we identified 90 upregulated and 94 downregulated metabolites (Supplementary Figure S16B). Notably, the overlap of differential metabolites between SMAnalyst and Cardinal reached 89% (Supplementary Figure S16C), demonstrating strong concordance in statistical findings while highlighting SMAnalyst’s advantages in producing more spatially coherent clusters and offering an integrated analytical workflow.

In addition to automated region selection based on clustering, SMAnalyst also supports manual definition of specific anatomical regions for targeted research. For example, by manually outlining the entorhinal cortex and primary visual cortex (Supplementary Figure S13: Primary visual cortex: regions 1 and 2; Entorhinal cortex: regions 3 and 4) and performing differential analysis, we successfully identified 9 significantly upregulated metabolites in the entorhinal cortex and 20 significantly upregulated metabolites in the primary visual cortex. This integrated analytical workflow fully demonstrates SMAnalyst’s powerful utility in flexibly addressing scientific research questions within a single environment.

### 3.5. Scalability and Performance Benchmarking

To address the scalability of SMAnalyst for handling large-scale spatial metabolomics datasets, we evaluated its performance across three datasets with progressively increasing dimensions, from 14,260 to 118,604 pixels (Supplementary Table S1). The platform demonstrated efficient processing on its web platform for datasets with tens of thousands of pixels; for instance, the 53,812-pixel mouse brain dataset was processed within 30 min. To validate performance on even larger scales, we deployed SMAnalyst locally on a standard workstation. The results show that the high-resolution dataset (118,604 pixels, 2898 ions) completed a full analysis workflow in 94 min with 33.92 GB of memory. Upload times for the peak tables were practical, ranging from 0.4 min for the smallest dataset to 5.9 min for the largest. These benchmarks confirm SMAnalyst’s capability to handle datasets exceeding 100,000 pixels within reasonable timeframes. For routine analysis, the web platform is sufficient for most datasets, while for extremely large datasets or to leverage greater computational resources, local deployment is recommended, for which the open-source code is freely available.

### 3.6. Impact of Noise Filtering on Spatial Clustering Across Datasets

To evaluate the impact of noise ion filtering on spatial clustering and address the need for validation across diverse datasets, we analyzed two mouse brain datasets with different spatial resolutions under varying noise filtering thresholds.

For Dataset 1, we performed UMAP-kmeans clustering under three conditions: (1) no noise filtering, (2) filtering at the default noise score threshold of 30, and (3) filtering at a stringent threshold of 60. The results demonstrate that noise filtering significantly improves clustering quality. Compared to the unfiltered data, filtering at threshold 30 produced more coherent and anatomically precise clusters in hippocampal and midbrain regions (Supplementary Figure S17A,B). The stringent threshold 60 provided further refinement but with diminishing returns (Supplementary Figure S17C).

Similar effects were observed in Dataset 2, which exhibited slightly higher noise levels with 41% of ions filtered at threshold 30. In this higher-resolution dataset, unfiltered clustering showed fragmented clusters with scattered patterns in midbrain regions (Supplementary Figure S18A). After noise filtering, these discrete clusters consolidated into more continuous, anatomically relevant regions (Supplementary Figure S18B,C), demonstrating that noise removal enhances cluster coherence and better aligns with tissue architecture across different dataset characteristics.

These results across two distinct datasets confirm that noise ion filtering consistently improves spatial clustering quality and that the default threshold of 30 provides a robust balance between noise removal and signal retention for diverse spatial metabolomics datasets.

## 4. Conclusions

SMAnalyst, as an innovative open-source platform, offers the first complete solution for spatial metabolomics research, integrating data quality control, preprocessing, spatial pattern analysis, differential comparison, and metabolite annotation. Its primary contribution to the MSI data analysis field is addressing the critical bottleneck of workflow fragmentation by offering a unified, web-based environment that eliminates the need for tool switching. Its core value lies in: (i) pioneering a multi-dimensional systematic data quality assessment; (ii) providing metabolite annotation based on multi-evidence scoring; and (iii) complementary spatial pattern discovery through both metabolite co-expression and pixel clustering perspectives. Despite these advances, we acknowledge certain limitations in the current implementation. As a cloud platform, SMAnalyst requires preprocessed feature matrices as input and does not support computationally intensive upstream processes like peak picking within its web interface. This design choice prioritizes analytical accessibility and performance for the core downstream workflow. Additionally, while SMAnalyst integrates multiple clustering methods, the biological interpretation of resulting spatial patterns still requires expert knowledge. The open-source nature of the tool ensures its extensibility and potential for community-driven development. Future versions will focus on integrating more clustering algorithms and pattern recognition methods, addressing batch effects in multi-section analysis, and incorporating more suitable differential analysis methods to continuously meet the evolving analytical needs of the spatial metabolomics field.

## Figures and Tables

**Table 1:** Comparison of Common Spatial Metabolomics Software.

Category	Specific Comparison Item	SMAnalyst	Cardinal [12]	MassImager [14]	MsiReader [15]	METASPACE [22]	Multi-MSIProcessor [25]	M2aia [26]	SmartGate [13]
Visualization	Single-ion Imaging	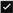	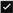	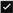	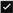	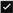	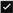	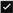	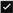
	Colocalization Analysis	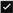	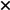	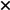	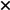	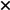	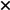	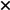	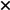
	Multi-ion Imaging	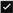	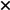	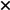	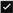	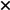	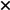	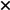	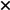
Quality Control	Background Consistency	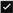	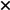	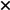	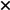	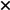	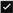	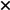	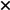
	Intensity	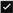	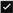	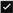	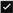	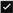	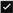	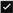	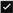
	Missing Values	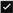	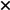	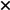	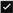	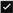	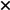	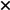	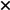
	Noise Ions	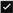	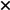	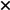	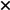	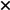	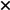	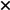	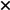
Pattern Analysis	Pixel Clustering Patterns	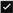	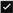	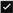	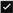	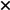	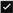	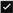	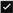
	Ion Spatial Expression Patterns	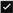	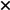	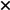	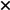	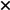	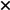	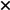	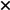
Differential Analysis	Differential Analysis Based on Manual Region Selection	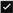	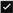	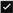	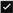	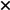	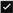	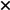	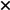
	Differential Analysis Based on Clustered Regions	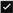	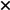	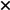	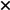	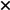	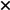	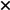	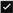
Metabolite Identification	Isotope Recognition	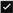	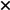	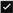	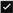	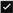	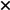	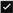	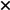
	Adduct Ion Recognition	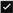	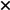	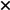	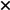	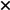	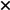	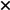	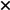
	Identification Result Scoring	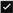	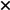	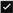	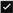	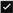	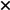	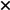	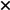
Others	Open Source	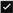	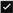	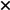	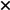	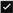	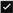	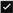	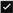
	Graphical User Interface (GUI)	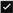	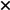	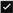	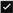	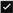	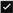	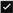	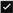
	Year of Last Update	2025	2023	2024	2018	2016	2023	2021	2023

## Data Availability

All resources described in this study are publicly available. The raw spatial metabolomics data and processed peak intensity tables have been deposited in the OMIX database of the National Genomics Data Center. The dataset1 is available under accession number OMIX011615 (https://ngdc.cncb.ac.cn/omix/release/OMIX011615, accessed on 27 August 2025), the dataset2 is available under accession number OMIX009541 (https://ngdc.cncb.ac.cn/omix/release/OMIX009541, accessed on 23 October 2025), while the dataset3 is accessible via accession number OMIX010192 (https://ngdc.cncb.ac.cn/omix/release/OMIX010192, accessed on 23 October 2025). The LC-MS/MS raw data are accessible via accession number OMIX011615 (https://ngdc.cncb.ac.cn/omix/release/OMIX011616, accessed on 27 August 2025).

## Supplementary Materials

The following supporting information can be downloaded at: https://www.mdpi.com/article/10.3390/biom15111562/s1, Table S1: Computational Performance of SMAnalyst Across Datasets of Varying Scales; Figure S1: Data Upload Format Requirements for SMAnalyst Software; Figure S2: SMAnalyst Tutorial Interface; Figure S3: Data Upload and Visualization Interface; Figure S4: QC1. Background Region Consistency Interface; Figure S5: Process1. Background Pixel Removal Interface; Figure S6: Process2. Noise Ion Proportion Interface; Figure S7: QC2&3. Signal Intensity and Missing Value Assessment Interface; Figure S8: Isotope Peak and Adduct Ion Peak Identification Interface; Figure S9: Format Requirements for Uploading Custom Library Files; Figure S10: Metabolite Identification Interface; Figure S11: Metabolite Spatial Pattern Analysis Interface; Figure S12: Spatial Metabolic Clustering and Cluster-Based Differential Analysis Interface; Figure S13: Differential Metabolic Analysis Interface Based on Manual Selection; Figure S14: Visualization Interface; Figure S15: Comparison of Metabolite Annotation Results Between SMAnalyst and MSIannotator; Figure S16: Comparison of Spatial Clustering and Differential Analysis Between SMAnalyst and Cardinal; Figure S17: Impact of Noise Filtering on Spatial Clustering in Dataset 1; Figure S18: Impact of Noise Filtering on Spatial Clustering in Dataset 2.

## Abbreviations

The following abbreviations are used in this manuscript:

SMAnalyst	Spatial Metabolomics Data Analyst
AFADESI	Air Flow-Assisted Desorption Electrospray Ionization
GUI	Graphical User Interface
ROI	Region of Interest
HIP	Hippocampus
MB	Midbrain
H&E	Hematoxylin and Eosin
