# Supplementary material for: SMAnalyst: A Web Server for Spatial Metabolomic Data Analysis and Annotation
Source: Biomolecules. 2025 Nov 6;15(11):1562. doi: 10.3390/biom15111562 (PMC12650421; doi:10.3390/biom15111562)

SMAnalyst: a web server for spatial metabolomic data analysis and annotation

Zhanlong Mei [1#*], Xiaolian Ning [2#], Haoke Deng [1], Lingyun Chen [2], Yun Zhao [1] and Jin Zi [1,*]

[1] BGI Genomics, Shenzhen 518083, China
[2] BGI Research, Shenzhen 518083, China
* *Jin Zi (zij@genomics.cn); Zhanlong Mei (meizhanlong@genomics.cn)*
\# These authors contributed equally to this work.

Table S 1 Computational performance of SMAnalyst across datasets of varying scales. 3

Figure S1 Data upload format requirements for SMAnalyst software 4
Figure S 2 SMAnalyst tutorial interface 5
Figure S 3 Data upload and visualization interface 6
Figure S 4 QC1: background region consistency interface 7
Figure S 5 Process1: background pixel removal interface 8
Figure S 6 Process 2: noise ion proportion interface 9
Figure S 7 QC2&3: signal intensity and missing value assessment interface 10
Figure S 8 Isotope peak and adduct ion peak identification interface 11
Figure S 9 Format requirements for uploading custom library files to SMAnalyst. 12
Figure S 10 Metabolite identification interface 13
Figure S 11 Metabolite spatial pattern analysis interface 14
Figure S 12 Spatial metabolic clustering and cluster-based differential analysis interface 15
Figure S 13 Differential metabolic analysis interface based on manual selection 16
Figure S 14 Visualization interface 17
Figure S 15 Comparison of metabolite annotation results between SMAnalyst and MSIannotator. (A) Proportion of ion peaks with annotation results identified by MSIannotator. (B) Distribution of one-to-many matching results for ion peaks in MSIannotator. (C) Overlap of ions with single annotation results between SMAnalyst and MSIannotator 18
Figure S 16 Comparison of spatial clustering and differential metabolite analysis between Cardinal and SMAnalyst. (A) Spatial clustering of the mouse brain dataset using Cardinal's spatial shrunken centroids method. (B) Differential metabolite analysis between midbrain (clusters 22 & 7) and hippocampal (cluster 3) regions identified by Cardinal. (C) Overlap of differential metabolites detected by Cardinal and SMAnalyst. 19
Figure S 17 Impact of noise filtering on spatial clustering in Dataset 1. (A) UMAP-kmeans clustering without noise filtering. (B) Clustering with noise filtering at threshold 30. (C) Clustering with noise filtering at threshold 60. 20
Figure S 18 Impact of noise filtering on spatial clustering in Dataset 2. (A) UMAP-kmeans clustering without noise filtering. (B) Clustering with noise filtering at threshold 30. (C) Clustering with noise filtering at threshold 60. 21

Table S 1 Computational performance of SMAnalyst across datasets of varying scales.

Dataset	Dataset1	Dataset2	Dataset3
Spatial Resolution/μm	100	50	20
Pixel Number	14,260	53,812	118,604
Ion Number	3,044	2,654	2,898
Raw data size/GB	8.8	20.8	77
Peak table size/MB	174	714.3	1700
Execution Environment	Web platform	Web platform	Local workstation
Upload time/min	0.4	0.8	5.9
Max Memory Usage/GB	4.87	7.59	33.92
Runtime/min	21	30	94

Figure S1 Data upload format requirements for SMAnalyst software

X	Y	89.023	90.0264	90.9699	91.0249	92.9266	94.8498	94.9236	94.9792	96.9634
59	89	3039486	74873	0	0	0	0	0	0	99058
59	90	2995561	0	0	0	0	0	0	0	0
59	91	2871624	0	0	0	0	0	0	0	0
59	92	3014818	0	0	0	0	0	0	0	0
59	93	3268235	0	0	0	0	0	0	0	92854
59	94	3614806	106503	0	0	0	0	0	0	119773
59	95	3543670	94454	0	0	0	0	0	0	95148
59	96	3400400	0	0	0	0	0	0	0	0
59	97	3061814	0	0	0	0	0	0	0	0
59	98	3285577	0	0	0	0	0	0	0	0
59	99	2832691	0	0	0	0	0	0	0	0
59	100	3374764	0	0	0	0	0	0	0	0
59	101	3107881	0	0	0	0	0	0	0	0
59	102	3020464	0	0	0	0	0	0	0	0
59	103	3212108	0	0	0	0	0	0	0	0
59	104	3617037	0	0	0	0	0	0	0	0
59	105	3836272	129136	0	0	0	0	0	0	0
59	106	3356250	0	0	0	0	0	0	0	0
59	107	3131629	0	0	0	0	0	0	0	0
59	108	3363901	0	0	0	0	0	0	0	0
59	109	3301902	0	0	0	0	0	0	0	0
59	110	3774631	0	0	0	0	0	0	0	0
59	111	3717910	0	0	0	0	0	0	0	0
59	112	3377615	0	0	0	0	0	0	0	0
59	113	3318233	0	0	0	0	0	0	0	0
59	114	3494989	0	0	0	0	0	0	0	0
59	115	3451147	0	0	0	0	0	0	0	0
59	116	3573527	0	0	0	0	0	0	0	0
59	117	3550325	0	0	0	0	0	0	0	0

Figure S 2 SMAnalyst tutorial interface

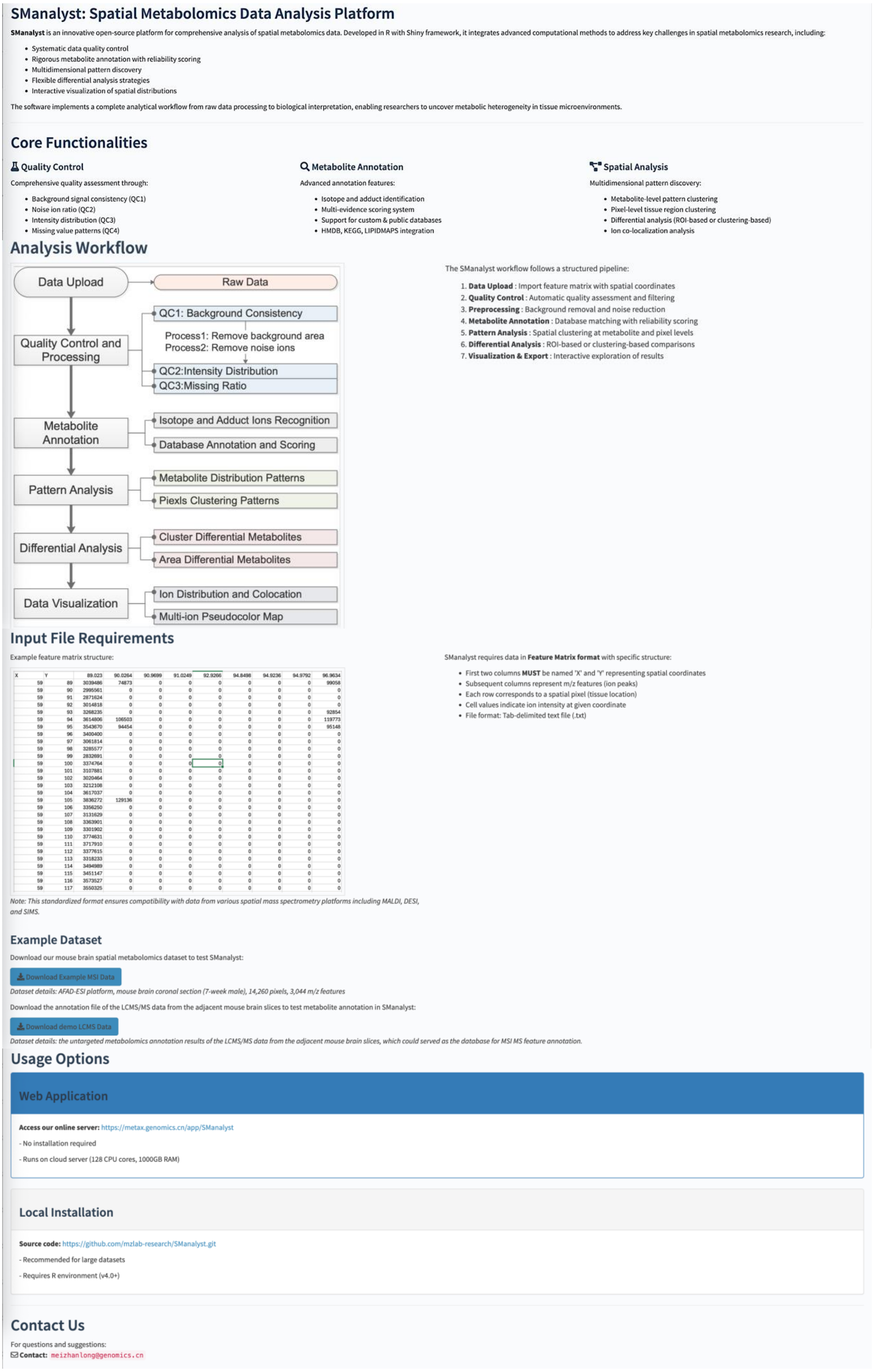

Figure S 3 Data upload and visualization interface

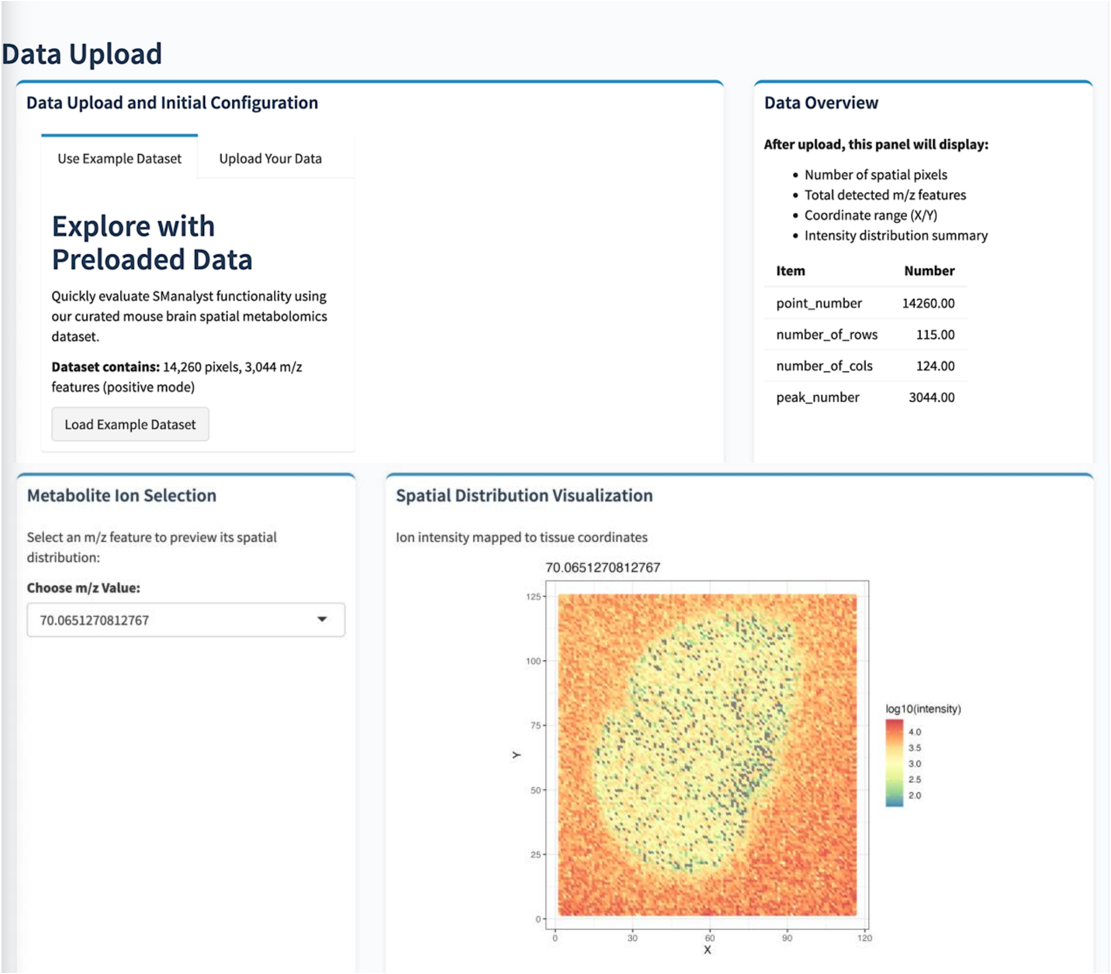

Figure S 4 QC1: background region consistency interface

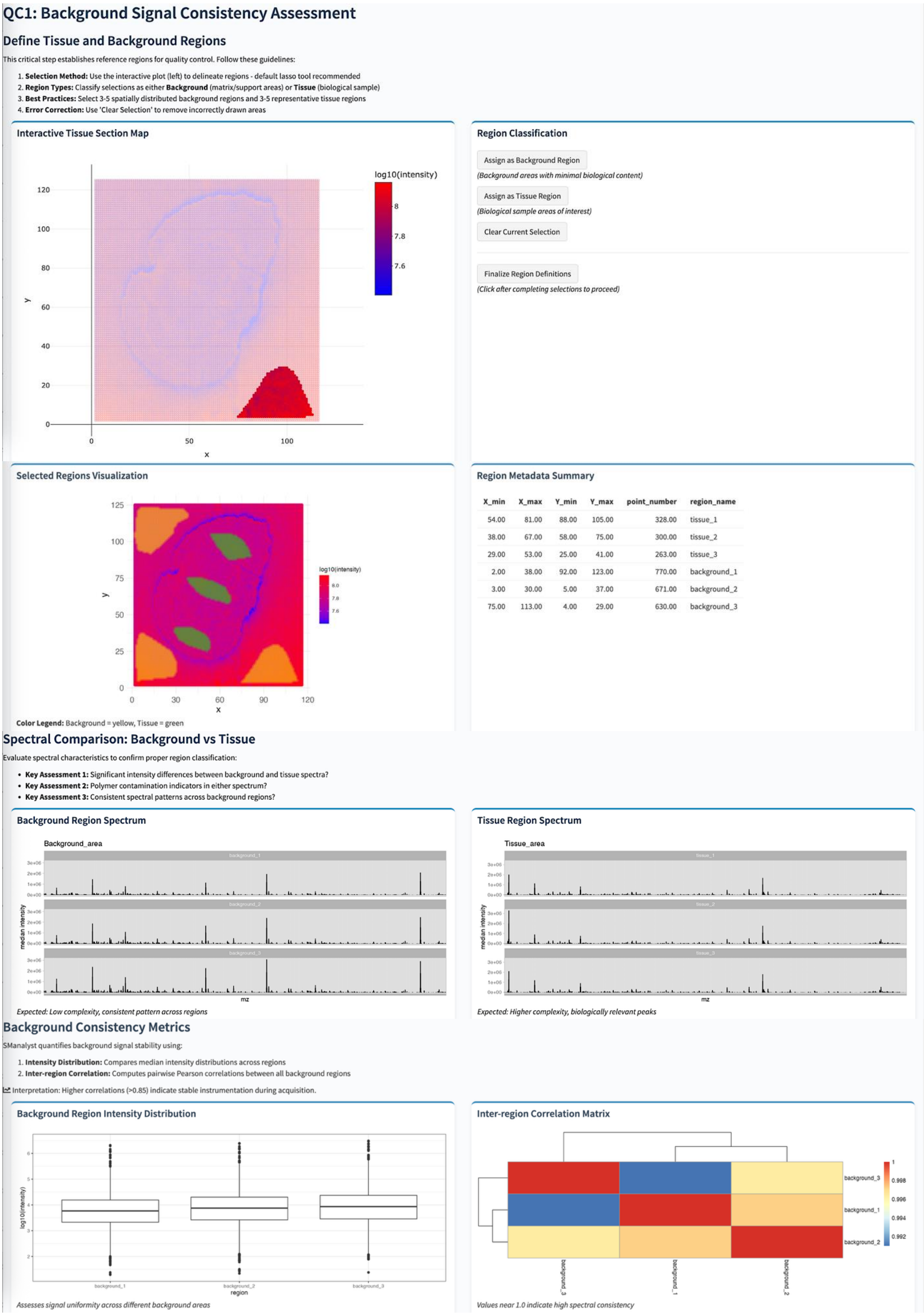

Figure S 5 Process1: background pixel removal interface

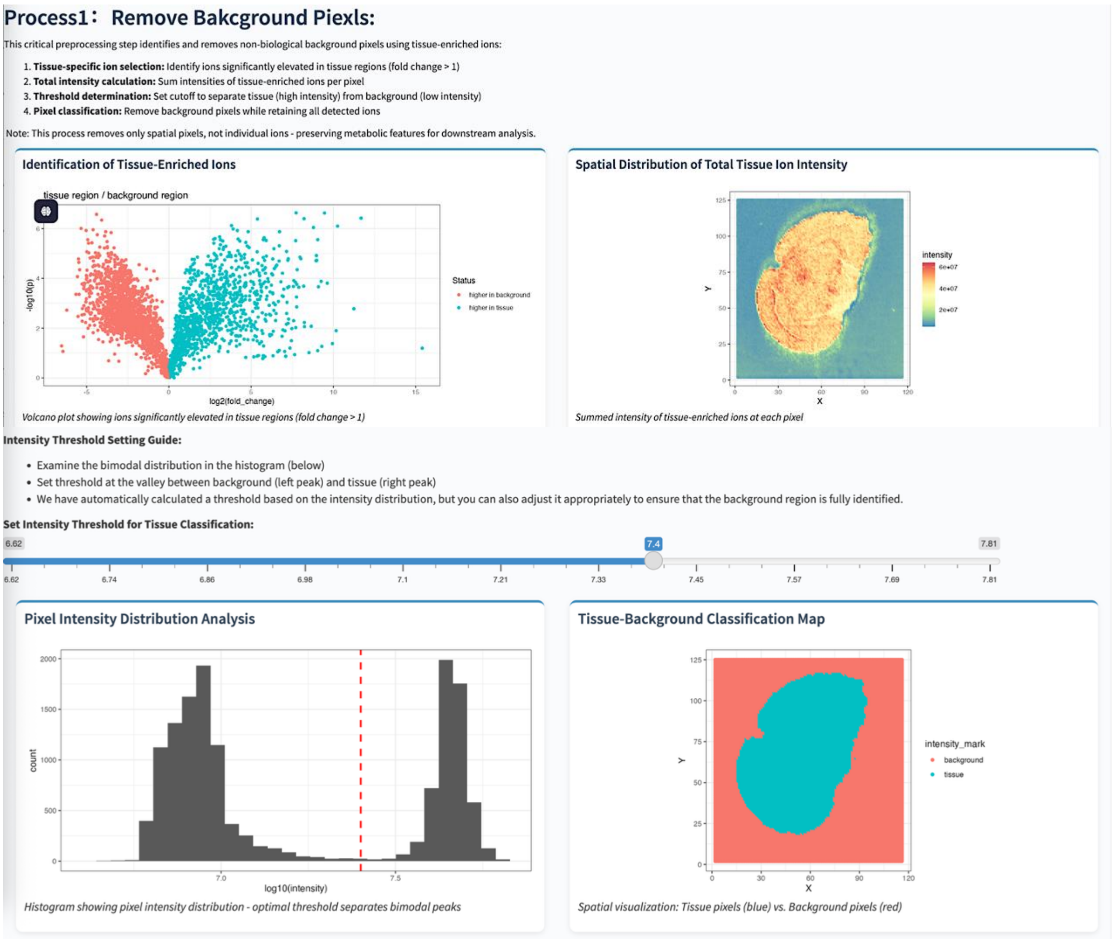

Figure S 6 Process 2: noise ion proportion interface

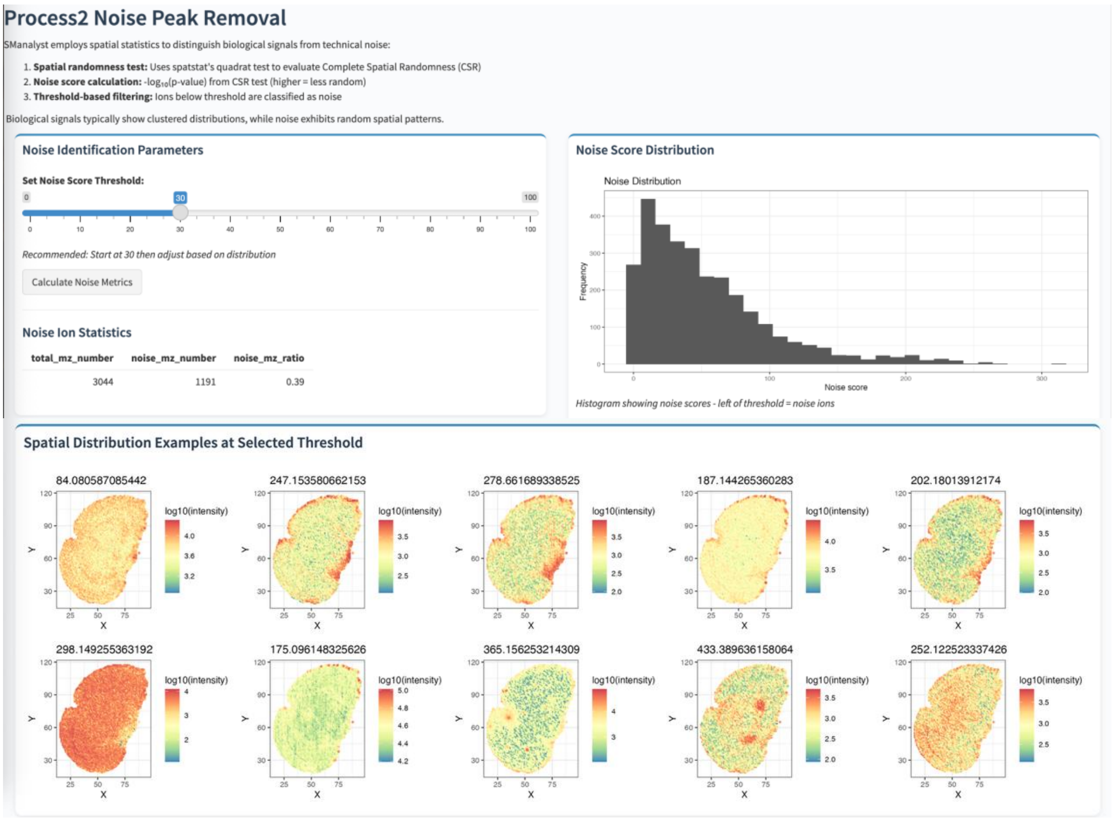

Figure S 7 QC2&3: signal intensity and missing value assessment interface

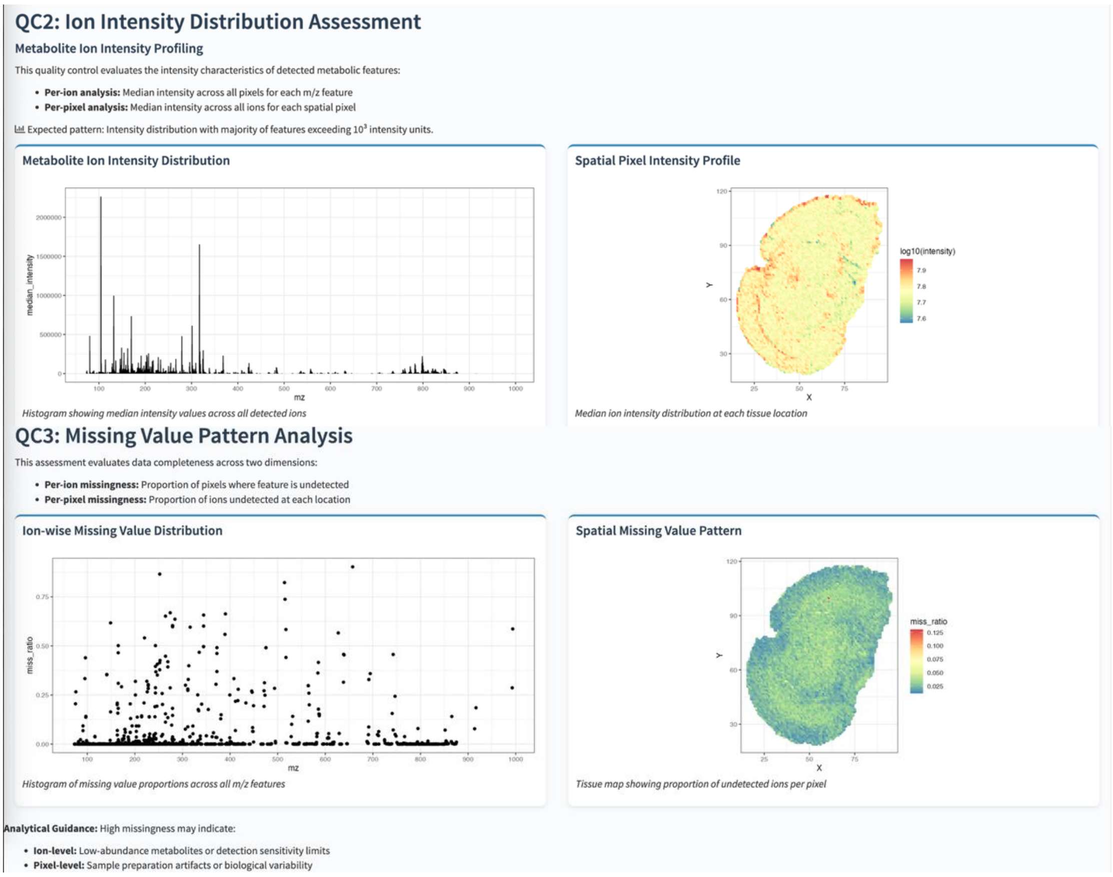

Figure S 8 Isotope peak and adduct ion peak identification interface

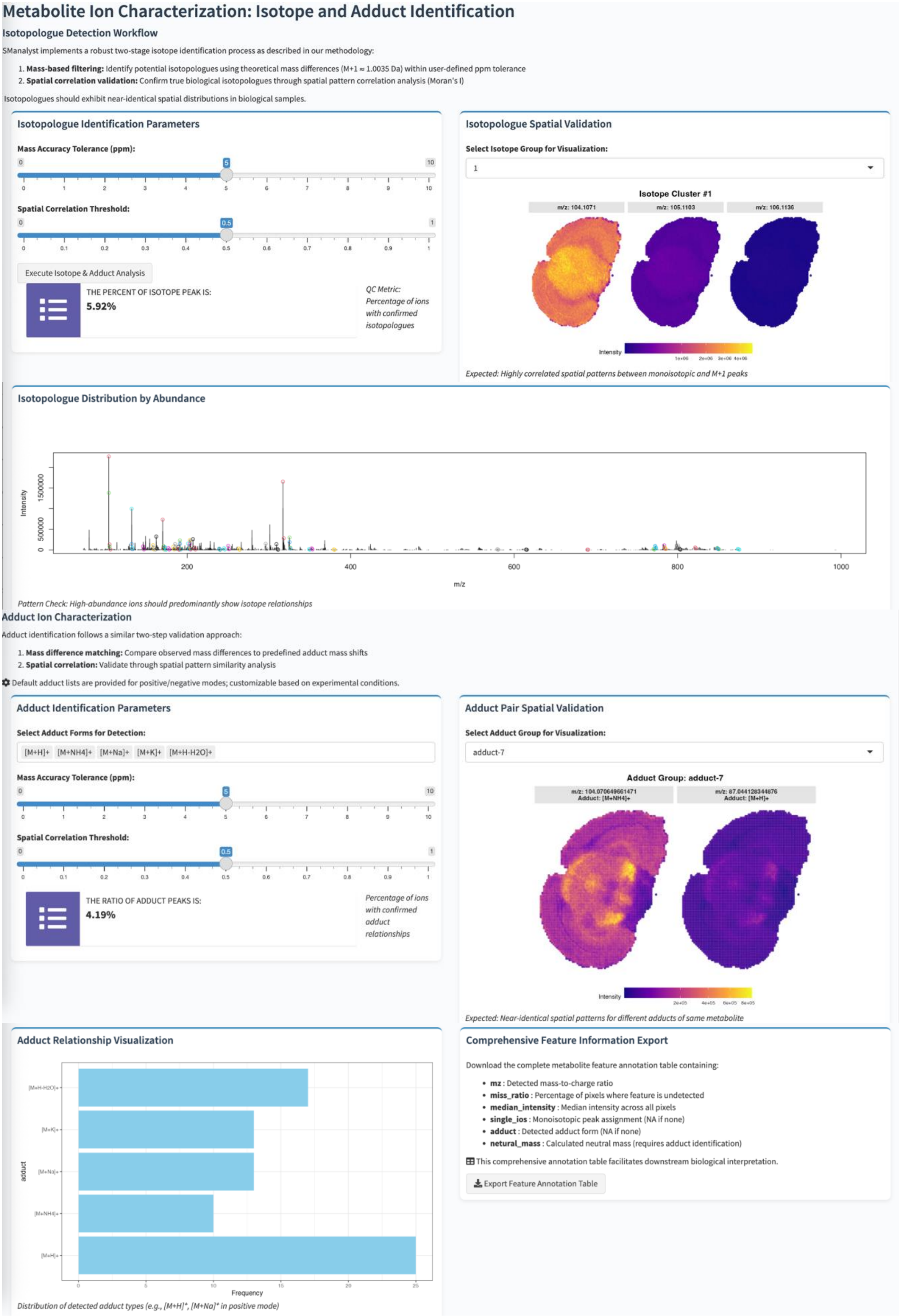

Figure S 9 Format requirements for uploading custom library files to SMAnalyst.

MW	Name	Formula	KEGG	HMDB	LIPIDMAPS
169.0851266	1-Methylhistidine	C7H11N3O2	C01152	HMDB0000001	
74.08439833	1,3-Diaminopropane	C3H10N2	C00986	HMDB0000002	
102.0316941	2-Ketobutyric acid	C4H6O3	C00109	HMDB0000005	LMFA01060002
104.0473441	2-Hydroxybutyric acid	C4H8O3	C05984	HMDB0000008	LMFA01050342
300.1725446	2-Methoxyestrone	C19H24O3	C05299	HMDB0000010	LMST02010033
104.0473441	3-Hydroxybutyric acid	C4H8O3	C01089	HMDB0000011	LMFA01050243
228.0746215	Deoxyuridine	C9H12N2O5	C00526	HMDB0000012	
227.0906059	Deoxycytidine	C9H13N3O4	C00881	HMDB0000014	
346.2144094	Cortexolone	C21H30O4	C05488	HMDB0000015	LMST02030086
330.2194948	Deoxycorticosterone	C21H30O3	C03205	HMDB0000016	LMST02030087
183.0531578	4-Pyridoxic acid	C8H9NO4	C00847	HMDB0000017	
116.0473441	alpha-Ketoisovaleric ac	C5H8O3	C00141	HMDB0000019	LMFA01020274
152.0473441	p-Hydroxyphenylacetic	C8H8O3	C00642	HMDB0000020	
306.9705366	Iodotyrosine	C9H10INO3	C02515	HMDB0000021	
167.0946287	3-Methoxytyramine	C9H13NO2	C05587	HMDB0000022	
104.0473441	(S)-3-Hydroxyisobutyri	C4H8O3	C06001	HMDB0000023	
891.646934	3-O-Sulfogalactosylcer	C48H93NO11S		HMDB0000024	
132.0534921	Ureidopropionic acid	C4H8N2O3	C02642	HMDB0000026	
241.1174894	Tetrahydrobiopterin	C9H15N5O3	C00272	HMDB0000027	
244.0881631	Biotin	C10H16N2O3S	C00120	HMDB0000030	
290.2245802	Androsterone	C19H30O2	C00523	HMDB0000031	LMST02020001
384.339216	7-Dehydrocholesterol	C27H44O	C01164	HMDB0000032	LMST01010069
226.1065903	Carnosine	C9H14N4O3	C00386	HMDB0000033	
135.0544952	Adenine	C5H5N5	C00147	HMDB0000034	
515.2916735	Taurocholic acid	C26H45NO7S	C05122	HMDB0000036	LMST05040001
360.193674	Aldosterone	C21H28O5	C01780	HMDB0000037	LMST02030026

Figure S 10 Metabolite identification interface

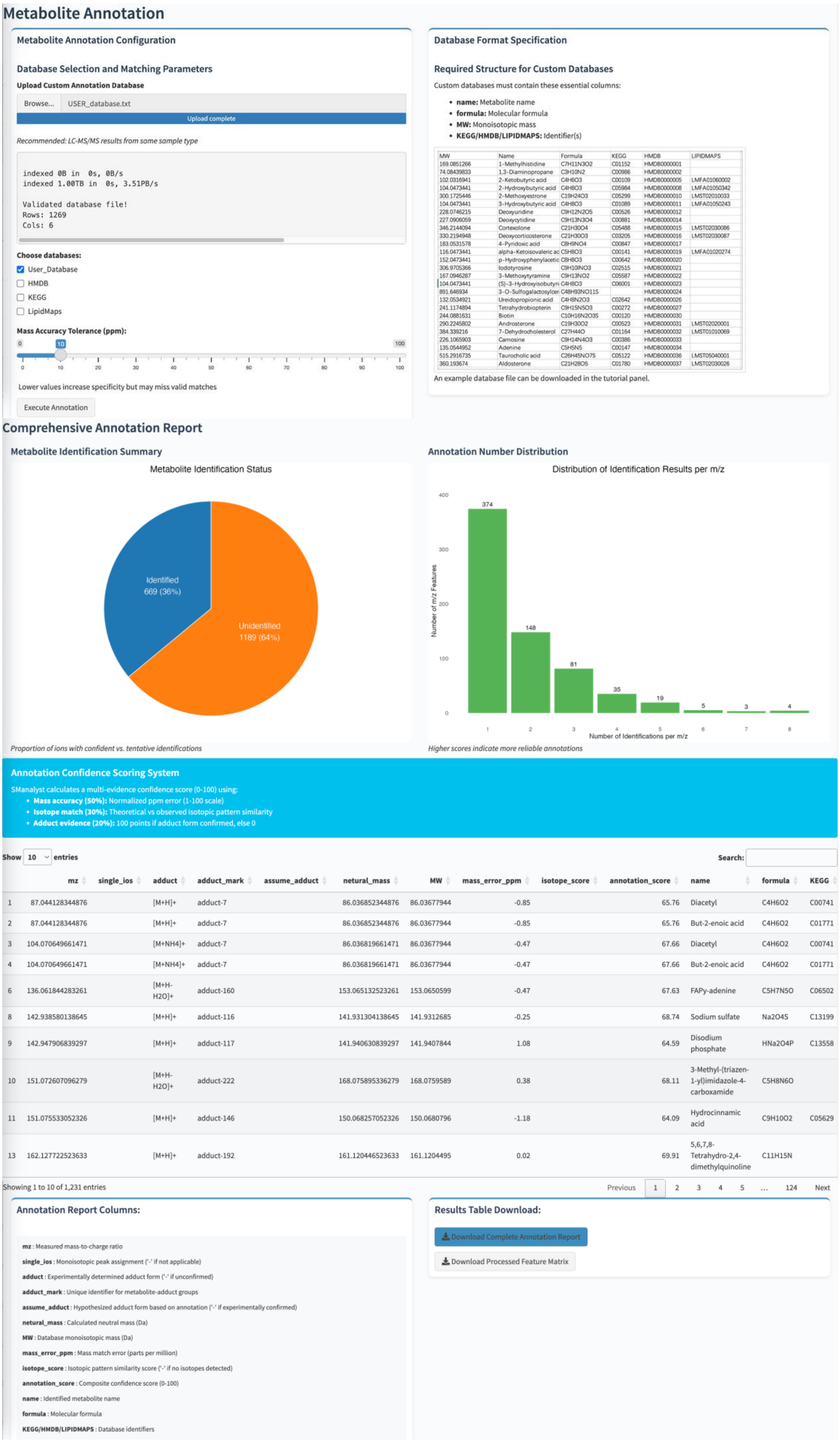

Figure S 11 Metabolite spatial pattern analysis interface

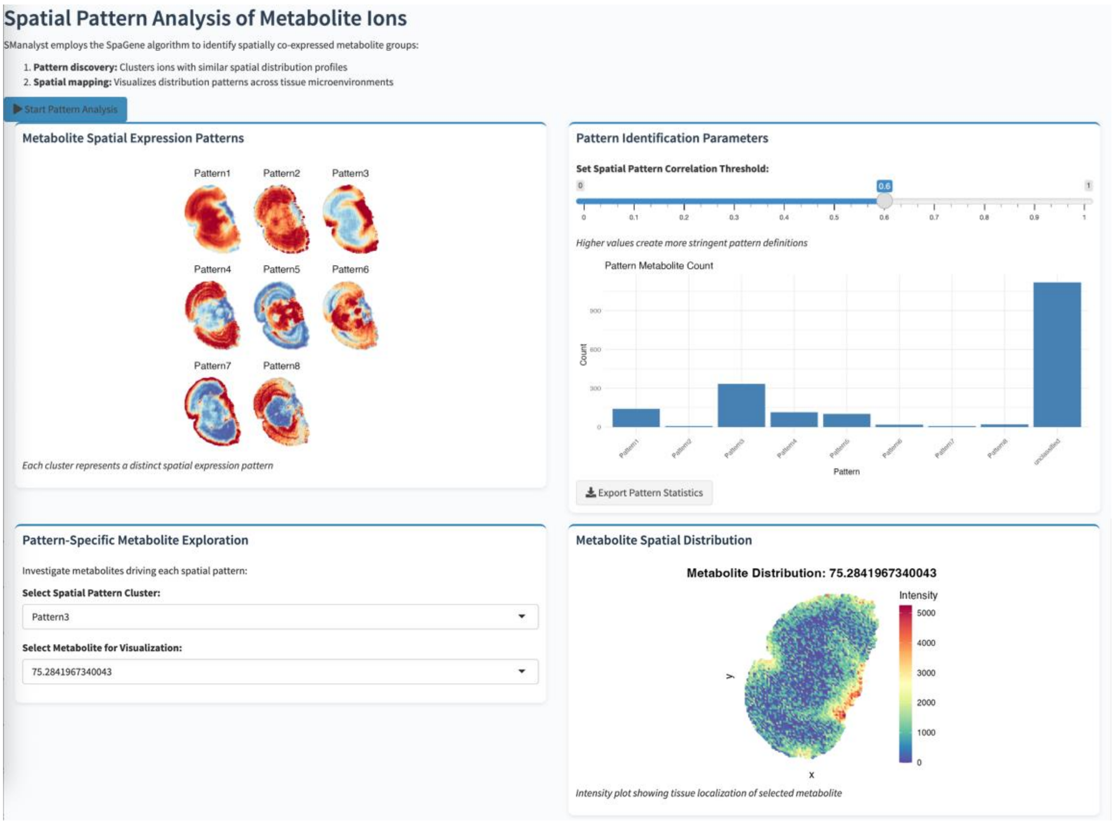

Figure S 12 Spatial metabolic clustering and cluster-based differential analysis interface

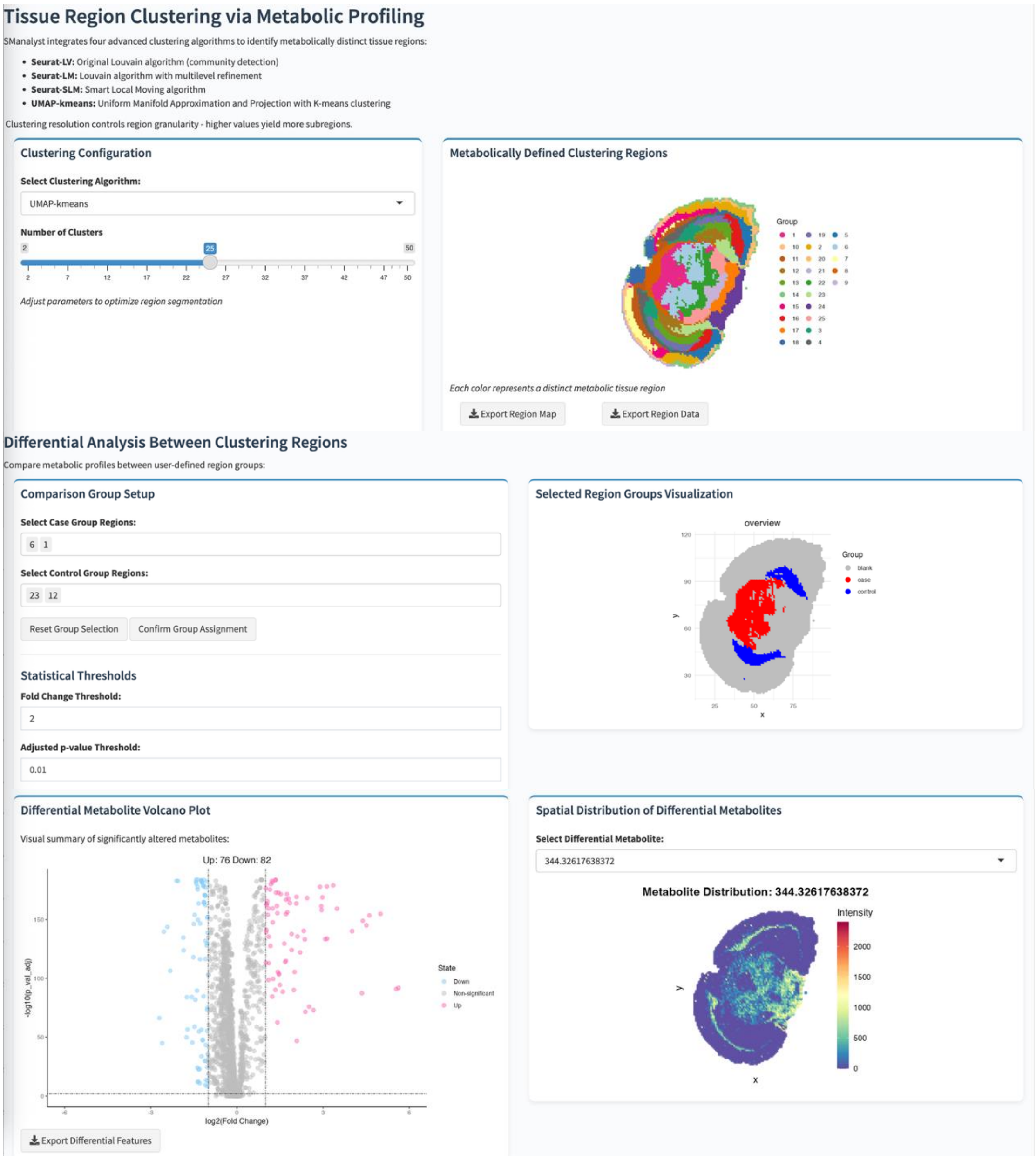

Figure S 13 Differential metabolic analysis interface based on manual selection

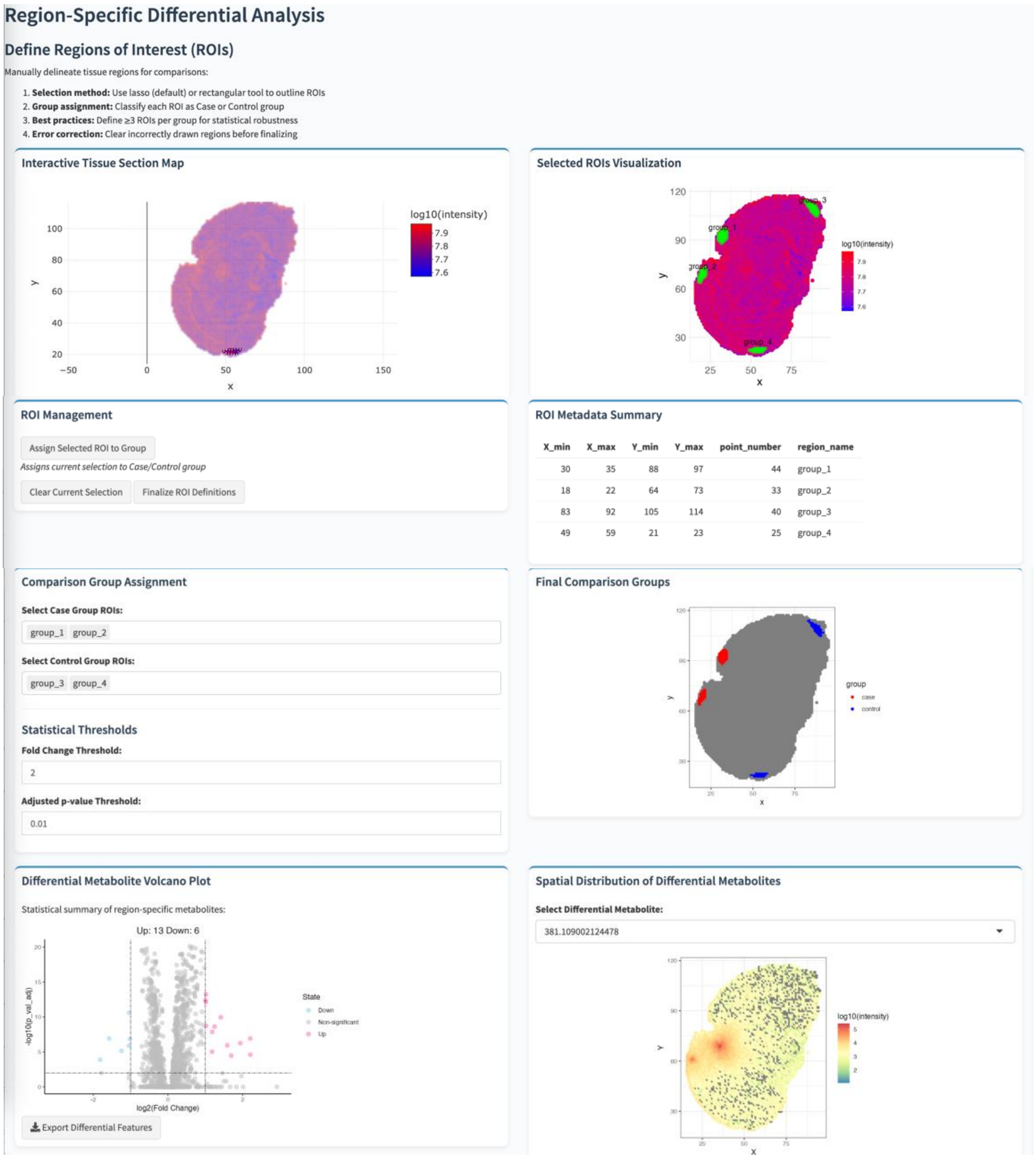

Figure S 14 Visualization interface

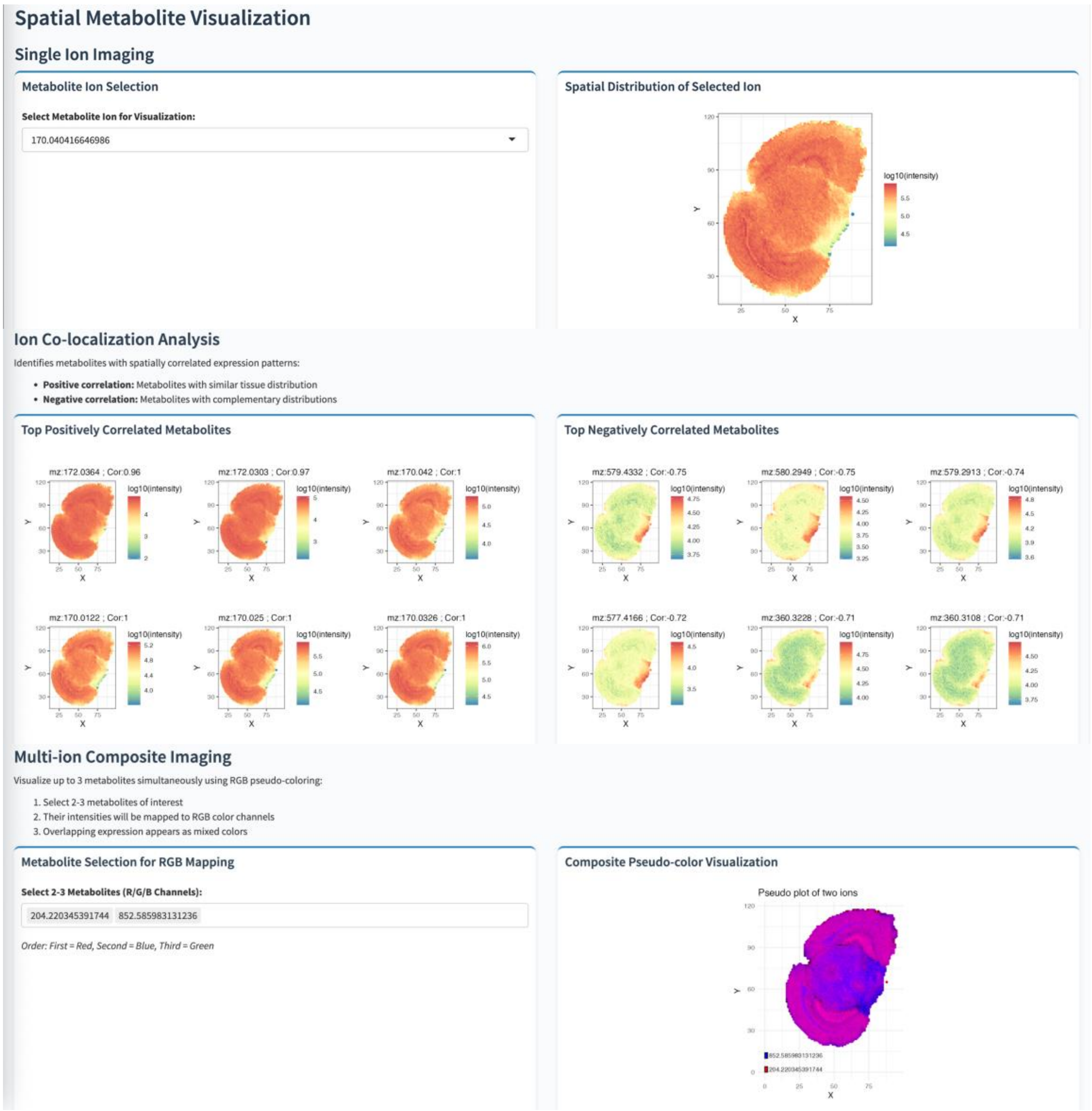

Figure S 15 Comparison of metabolite annotation results between SMAnalyst and MSIannotator. (A) Proportion of ion peaks with annotation results identified by MSIannotator. (B) Distribution of one-to-many matching results for ion peaks in MSIannotator. (C) Overlap of ions with single annotation results between SMAnalyst and MSIannotator

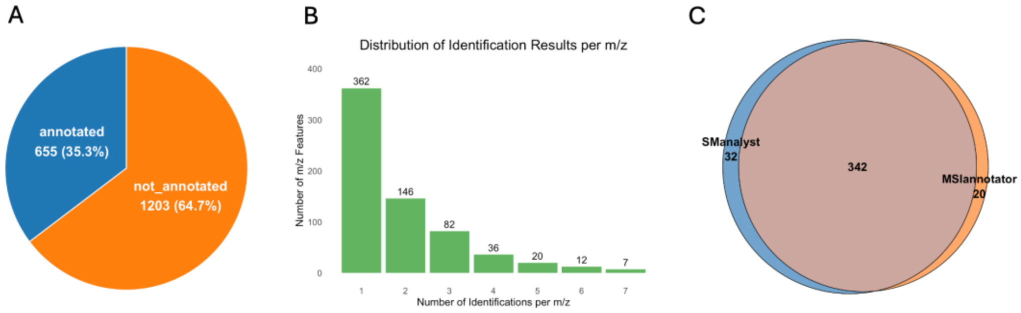

Figure S 16 Comparison of spatial clustering and differential metabolite analysis between Cardinal and SMAnalyst. (A) Spatial clustering of the mouse brain dataset using Cardinal’s spatial shrunken centroids method. (B) Differential metabolite analysis between midbrain (clusters 22 & 7) and hippocampal (cluster 3) regions identified by Cardinal. (C) Overlap of differential metabolites detected by Cardinal and SMAnalyst.

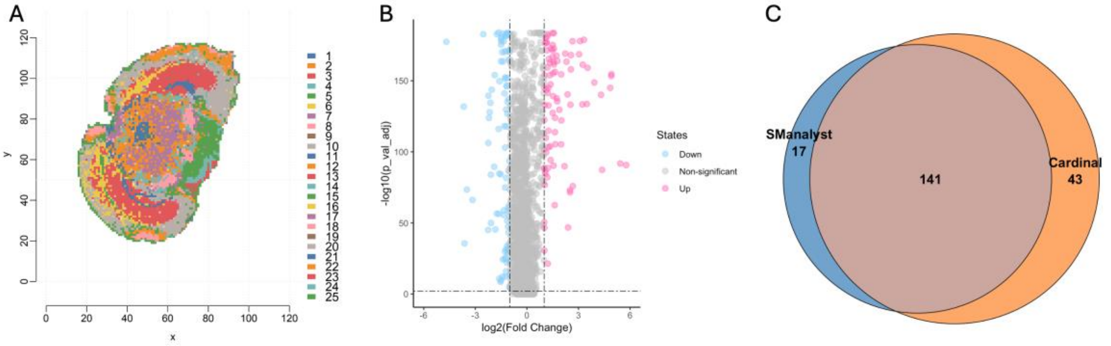

Figure S 17 Impact of noise filtering on spatial clustering in Dataset 1. (A) UMAP-kmeans clustering without noise filtering. (B) Clustering with noise filtering at threshold 30. (C) Clustering with noise filtering at threshold 60.

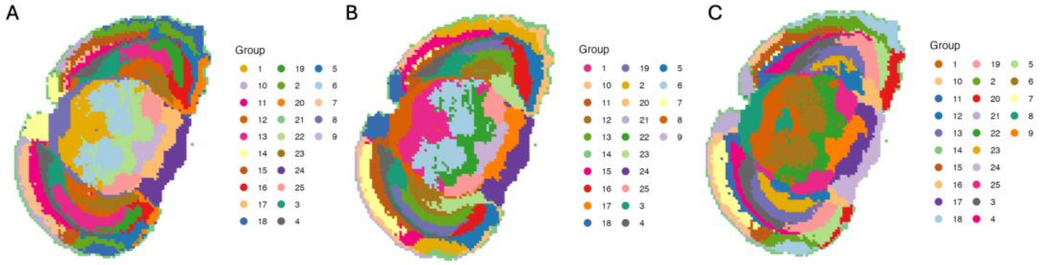

Figure S 18 Impact of noise filtering on spatial clustering in Dataset 2. (A) UMAP-kmeans clustering without noise filtering. (B) Clustering with noise filtering at threshold 30. (C) Clustering with noise filtering at threshold 60.

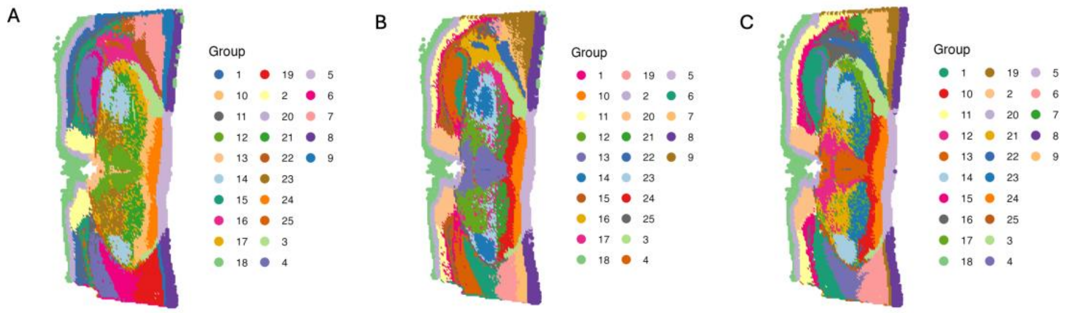

Supplement: Supplementary file 1 [file biomolecules-15-01562-s001.zip › biomolecules-3911418-supplementary.pdf]
